# Differential expression of miRNA in rat myocardial tissues under psychological and physical stress

**DOI:** 10.3892/etm.2014.1504

**Published:** 2014-01-27

**Authors:** YAN CUI, YUN BAI, XIAO DONG WANG, BIN LIU, ZHUO ZHAO, LI SHUANG WANG

**Affiliations:** 1 Department of Cardiology, The Second Hospital of Jilin University, Changchun, Jilin 130041, P.R. China; 2 Institute of Genetics and Cytology, School of Life Sciences, Northeast Normal University, Changchun, Jilin 130024, P.R. China; 3 Department of Endoscopy, The Second Hospital of Jilin University, Changchun, Jilin 130041, P.R. China; 4 Department of Cardiology, The General Hospital of CNPC in Jilin, Jilin, Jilin 132022, P.R. China

**Keywords:** stress, microRNAs, myocardium, rat, microarray

## Abstract

In the current study, microRNA (miRNA) microarrays were used to detect differentially expressed miRNAs in the myocardial tissues of rat models under stress, to screen target miRNA candidates for miRNA therapy of stress-induced myocardial injury. Rats were bound and suspended in order to induce acute stress (AS) and chronic stress (CS) models. miRNA microarrays were used to detect differentially expressed miRNA in the myocardial tissues of the stressed and control groups. In comparison to the normal control, there were 68 differentially expressed miRNAs in the AS model, of which 32 were upregulated and 36 were downregulated. There were 55 differentially expressed miRNAs in the CS model, of which 20 were upregulated and 35 were downregulated. Of the 123 miRNAs, 15 miRNAs were differentially expressed between the AS and CS groups, of which four were significantly upregulated (rno-miR-296, rno-miR-141, rno-miR-382 and rno-miR-219-5p) and 11 were downregulated (significantly downregulated, rno-miR-135a and rno-miR-466b). The stress of being bound and suspended induces myocardial injury in the rats. Myocardial injury may cause the differential expression of certain miRNAs. Psychological stress may lead to the significant upregulation of rno-miR-296, rno-miR-141, rno-miR-382 and rno-miR-219-5p in addition to the significant downregulation of miR-135a and miR-466b.

## Introduction

Stress is a non-specific systemic adaptive response of the body stimulated by a variety of internal and external environmental, social and psychological factors, also known as the stress response. Sympathetic and parasympathetic nervous system balance and hypothalamic-pituitary-adrenal (HPA) axis function alter under stress stimulation (1). Furthermore, external stimuli signals are delivered through multiple pathways or channels into the cells and trigger a series of cell reactions, including protein synthesis, degradation and cytokine secretion (2,3). These cause cardiomyocyte proliferation and hypertrophy or apoptosis, resulting in myocardial injury.

microRNA (miRNA) is small non-coding RNA of 18–26 bp. miRNA causes the degradation of, or inhibits the translation of, target mRNA by pairing with specific bases of the target mRNA and is thus involved in the post-transcriptional regulation of gene expression (4,5). Studies have observed that miRNA expression has temporal and tissue specificity (1–6). miRNA is important in various developmental stages of the cardiovascular system, regulating cardiomyocyte proliferation, differentiation and apoptosis under physiological and pathological conditions (7–19). Dozens of miRNAs have been identified in myocardial cells, including miR-133a, miR-133b, miR-1d, miR-296, miR-21, miR-208 and miR-195 (4–18). Studies have demonstrated that miRNA has an important role in the regulation of heart development, cardiac hypertrophy, cardiac electrophysiology and angiogenesis. The use of myocardial genomic research in the study of stress-induced myocardial injury may improve the understanding of the mechanisms of stress-induced myocardial injury. However, there are currently no reports on the role of miRNA underlying stress-induced myocardial injury.

In the present study, rat models of myocardial injury induced by psychological stress were established and miRNA microarrays were used to analyze the changes in the miRNA expression profiles of injured myocardial tissues. Furthermore, the role of miRNA in stress-induced myocardial injury was studied and an attempt was made to locate the target miRNAs which regulate stress-induced myocardial injury, which may provide the basis for the development of new drugs for the prevention and treatment of stress-induced myocardial injury.

## Materials and methods

### Animals

A total of 24 eight-week-old male Wistar rats with body weights of 220±10 g were supplied by the Experimental Animal Center of Jilin University Norman Bethune Medical Division (Changchun, China). The laboratory was disinfected, quiet and well-ventilated. The animals were provided with free access to food and water and were housed at a temperature of 22±2°C, with 50% humidity and a 12 h natural light-dark cycle. The rats were randomly divided into the normal control group (NC; n=8), the chronic stress group (CS; n=8) and the acute stress group (AS; n=8). The Jilin University Animal Ethics Committee approved the protocol for this study (no. 2012036).

### Modeling

The method for modeling stress in a rat has been described previously (21). For CS modeling, the limbs of the rat were bandaged to a board, with the tail kept free; subsequently the rats were hung upside down and denied access to food and water randomly for 2 h each day for 14 consecutive days. For AS modeling, the rats fasted for 12–24 h with access to water, and were subsequently hung upside down with their limbs bound to a board for 6 h. In all groups, blood was collected and the concentration of adrenocorticotropic hormone (ACTH) was determined using an ELISA kit (R&D, Minneapolis, MN, USA) in order to assess the stress state effect of the model.

Statistical processing for this section was performed as follows: the SPSS 16.0 software package (IBM Corp., Chicago, IL, USA) was used for statistical analysis and measurement data were stated as the mean ± standard deviation. Comparisons were performed using the mean adopted t-test, and P<0.01 was considered to indicate a statistically significant difference.

### Extraction and qualification of total RNA

The rats underwent thoracotomies under anesthesia in order to remove their hearts. The hearts were washed using 0.2 mol/l phosphate buffer saline (pH 7.0) and stored in liquid nitrogen. Next, ~100 mg myocardial tissues were frozen and crushed using a BioPulverizer™ (Aoran Technology LTD, Shanghai, China), mixed with 1 ml TRIzol reagent (Invitrogen Life Technologies, Carlsbad, CA, USA), and homogenized. Total RNA was extracted using the TRIzol reagent method. Extracted RNA was dissolved in RNAse-free water and incubated at 60°C for 10 min.

Absorbance at 260, 280 and 230 nm was measured using an ultraviolet spectrophotometer (ND-1000; NanoDrop, Wilmington, DE, USA). A_260_/A_280_ and A_260_/A_230_ ratios were calculated to determine the purity of RNA. The RNA concentration was calculated according to the formula: A_260_ × 40 ng/μl. Denaturing agarose gel electrophoresis was performed. Briefly, 3 μg RNA were samples stored in RNAse-free water mixed with formaldehyde-containing loading buffer (containing 10 μg/ml ethidium bromide) at a volume ratio of 1:3 and immediately incubated at 70°C for 15 min in order to denature the samples. Electrophoresis was performed at a voltage of 6 V/cm for 10–15 min, and gel images were captured using a gel image processing system (UVP EC3 600 Imaging System, UVP LLC, Upland, CA, USA).

### miRNA labeling

miRNA was labeled using the miRCURY™ Array Power Labeling kit (cat no. 208032-A; Exiqon, Inc., Woburn, MA, USA) in accordance with the manufacturer’s instructions. Briefly, 4 μl calf intestine phosphatase (CIP) reaction solution (1 μl total RNA, 0.5 μl CIP buffer, 0.5 μl CIP and 2 μl ddH_2_O) was incubated at 37°C for 30 min and then at 95°C for 1 min to terminate the reaction, and immediately placed in an ice bath for 10 min. Following mild centrifugation (Sigma 4K 15CR; 200 × g), 3 μl labeling buffer, 1.5 μl fluorescent labels (Hy3™ for the stress group or Hy5™ for the control), 2 μl DMSO and 2 μl labeling enzyme were sequentially added in an ice bath. The system was incubated at 16°C for 1 h and subsequently at 65°C for 15 min in order to terminate the labeling reaction, and stored at 4°C following mild centrifugation.

### miRNA microarray hybridization and scanning

The labeled RNAs and miRCURY Array chip (Shanghai Kangcheng BioEngineering Co., Ltd., Shanghai, China) were hybridized in accordance with the manufacturer’s instructions. Briefly, 180 μl reaction mixture (25 μl labeled miRNA, 90 μl 2X hybridization buffer and 65 μl nuclease-free buffer) was incubated at 95°C in the dark for 2 min and subsequently placed in an ice bath for 10 min. At the same time, the miRCURY Array chip was assembled according to the manufacturer’s instructions (Shanghai Kangcheng BioEngineering Co., Ltd.). The reaction mixture was loaded through the loading port and the 1X hybridization buffer was used to fill the hybridization chamber. The microarray chip was packaged in a protective bag and placed vertically into water (95°C) for 12 h, followed by drying in an oven at 56°C over night. The chip was washed using the miRCURY Array Wash Buffer kit (cat no. 208021, Exiqon, Inc., Woburn, MA, USA) according to the manufacturer’s instructions, followed by centrifugation at 200 × g for 5 min to dry the chip. The scanning procedure was performed using an Axon GenePix 4000B microarray scanner (Axon Instruments, Inc., Foster City, CA, USA) to obtain the scanning profiles, followed by data analysis and analysis of the significant difference using GenePix Pro6.0 software (Axon Instruments, Inc.).

### Data processing and analysis

An experienced operator utilized the GenePix Pro6.0 software for data processing and analysis. The signal points included in the subsequent analysis should conform to two prerequisites: i) The signal intensity of the red and green channels is >0; and ii) the signal to noise ratio (SNR) of the two channels is >1, or either SNR is >2. The weak signal points that did not conform to the two prerequisites were excluded from the subsequent analysis. miRNA was also excluded provided it contained 3 or 4 weak signal points in a total of four repeat points.

Lowess standardization (intra-chip standardization) was performed to remove dye intensity-dependent deviation. Scale standardization (inter-chip standardization) was used to reduce the experimental or sample errors between the different chips. Four repeat points in the chip were merged by calculating the median of the Hy5/Hy3 ratio of each point. Subsequently the differentially expressed miRNAs were determined using the statistical Student t-test. P<0.05 was considered to indicate a statistically significant difference. Fold change (stress/control) ≥1.5 indicated upregulation and <0.67 indicated downregulation.

## Results

### Modeling

**Figure 1:**
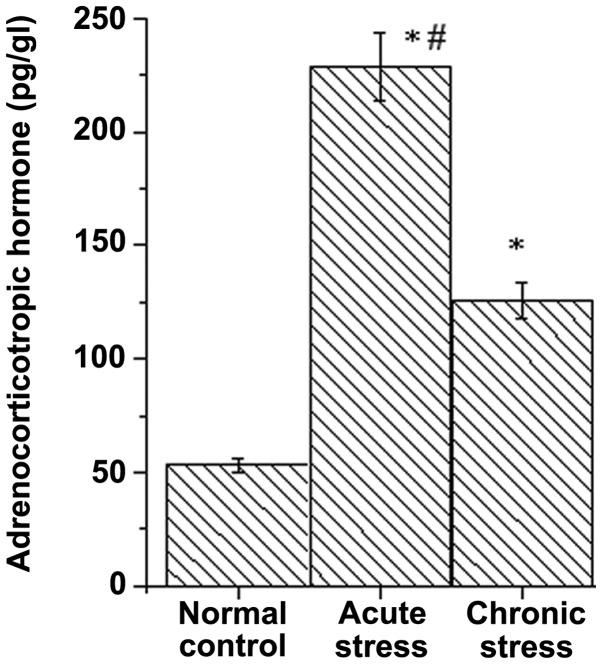
Adrenocorticotropic hormone concentrations in models/Data are presented as mean ± standard deviation (pg/ml; n=8). ^*^P<0.01, vs. normal control; ^#^P<0.05, chronic stress group.

Rats in the AS group appeared to be in a stressed state immediately following the surgery, but in the CS group, stress appeared after five days. The HPA system excites the stress stimulus and may be involved in abnormal secretion of corticotropin-releasing hormone (CRH), ACTH and three glucocorticoid hormones. The paraventricular nucleus of the hypothalamus secretes CRH under stress. The adenohypophysis CRH receptors perceive the change in ACTH and cause the adrenal cortex to secrete ACTH. ACTH is a key hormone of the HPA axis. Therefore, ACTH was selected to evaluate the stress state. As shown in Fig. 1, the ACTH levels of the stress model group increased significantly compared with the control group (P<0.01), indicating that the model was in a stressed state. ACTH levels of the AS model increased significantly compared with the CS model (P<0.05).

### Extraction and qualification of total RNA

**Figure 2:**
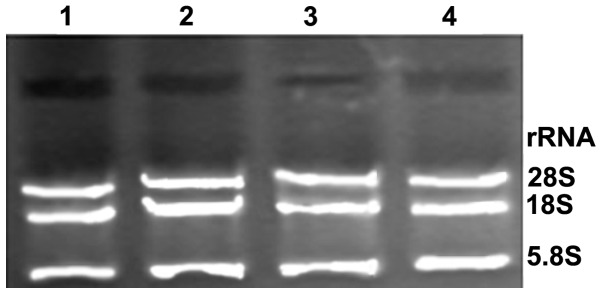
Ribosomal RNA separations on denaturing agarose gel. Lanes 1–4: NC1, NC2, acute stress group, chronic stress group, respectively. NC, normal control.

The A_260_/A_280_ ratio of RNA solution is a method for detecting RNA purity and values close to 2.0 are considered to represent pure RNA. A ratio <1.8 indicates sample contamination. A ratio >2.0 indicates RNA hydrolysis. The ratio range between 1.8 and 2.1 is acceptable. In addition, the A_260_/A_230_ ratio should be >1.8 for pure RNA. As demonstrated in Table I, the extracted RNAs conformed to the quality standards discussed and thus qualified for the subsequent miRNA experiments. On the denaturing gel, the 28S, 18S and 5.8S ribosomal RNA (rRNA) bands were bright (Fig. 2), and the rRNA bands demonstrated no signs of impurity. This indicated that the extracted total RNA was complete, RNA degradation and contamination were low, and the extracted total RNA exhibited high levels of purity and were of sufficient quality to qualify for use in subsequent miRNA experiments.

### Differential expression of miRNAs

According to data processing and analysis, specific miRNAs were identified to be differentially expressed in the stress model by comparing them with the normal control group (Tables II and III). There were 68 differentially expressed miRNAs in AS model, of these, 32 were upregulated and 36 were downregulated; there were 55 differentially expressed miRNAs in the CS model, of these, 20 were upregulated and 35 were downregulated. Of the 123 miRNAs, 15 were differentially expressed in the AS and CS model groups, of these, four were significantly upregulated (rno-miR-296, rno-miR-141, rno-miR-382 and rno-miR-219-5p; Table IV) and 11 were downregulated (significantly downregulated, rno-miR-135a and rno-miR-466b; Table V).

## Discussion

Excessive psychological stress leads to myocardial injury by changing the function of the sympathetic nervous system and the HPA axis. Studies have demonstrated that miRNA expression is tissue-specific and is involved in the formation and maintenance of tissue specificity during biological development (4–18). In myocardial cells numerous miRNAs have been identified to have an important role in the various developmental stages of the cardiovascular system by regulating cardiomyocyte proliferation, differentiation and apoptosis under physiological and pathological conditions (1–3).

The present study analyzed miRNA expression profiles in the myocardial tissues of AS and CS rat models using miRNA microarray technology and identified specific differentially expressed miRNAs. In the differentially expressed miRNAs of the AS and CS models, 15 miRNAs were differentially expressed in the AS and CS models. Of these 15 miRNAs, rno-miR-296, rno-miR-141, rno-miR-382 and rno-miR-219-5p were significantly upregulated, particularly miR-296 (Table IV), and 11 were downregulated (miR-135a and miR-466b were significantly downregulated, particularly miR-135a; Table V). These results indicate that miRNA changes caused by stress-induced myocardial injury are different from the miRNA changes caused by other factors. Therefore, the application of a reasonable stress-induced animal model, to explore the changes in the miRNAs of myocardial tissues under stress and identify specifically expressed miRNAs, is conducive to the further use of RNA interference for the treatment of stress-induced cardiovascular complications.

miR-125b, miR-146, miR-150, miR-199a, miR-21, miR-129, miR-341 and miR-451 have been confirmed to play an important role in the different developmental stages of the cardiovascular system (4–18). They are also significantly differentially expressed in the stress-induced model established in this study, indicating that these miRNAs may be common targets for myocardial injury caused by various factors. Stress can cause myocardial injury, thus leading to changes in these miRNAs. This finding also confirmed that stress itself is an important factor for myocardial injury.

In two stress-induced models, rno-miR-296 was the most significantly upregulated and miR-135a was the most significantly downregulated. Würdinger *et al* (19) observed that miR-296 may reduce the level of hepatocyte growth factor-regulated tyrosine kinase substrate (HGS) and induce the decrease in expression level of HGS-mediated vascular endothelial growth factor receptor-2 (VEGFR2) and platelet-derived growth factor receptor β by interacting with the substrate mRNA of target HGS. miR-296 promotes upregulation of VEGFR and contributes to angiogenesis. In addition, the inhibition of miR-296 has been found to reduce angiogenesis of xenograft tumors (19). In the present study, psychological stress-induced myocardial injury led to the upregulation of miR-296.

NR3C2 is a ligand-dependent transcription factor associated with steroid hormones. The transcription factor regulates the balance of water and ions and affects blood pressure by regulating water-sodium retention. Sõber *et al* (21) verified that NR3C2 may be the target gene of miR-135a and is involved in the regulation of blood pressure by inhibiting the *in vitro* translation of NR3C2 to regulate the angiotensin-aldosterone system balance. In the present study, miR-135a was significantly downregulated. We hypothesized that miR-135a may interact with the target genes to inhibit sympathetic nerve excitation and suppress the HPA axis and the renin-vascular angiotensin system, resulting in the release of a variety of stress hormones, including catecholamines, cortical hormone, pancreatic glucagon and renin, thus protecting the myocardium from injury.

In conclusion, rno-miR-296, rno-miR-141, rno-miR-382, rno-miR-219-5p, miR-135a and miR-466b may be involved in stress at the molecular level, thus causing myocardial injury. The development of stress-induced myocardial injury is a complex biological process and involves a variety of mechanisms. After cells receive external stimuli, stimulatory signals are transferred through multiple pathways or channels into the cells, causing a series of reactions. Altering the conditions in these cells may lead to the activation of the cell death pathway, particularly the activation of the mitochondrial death mechanism, causing a death cascade reaction, which includes cell necrosis and apoptosis. miRNA may cause degradation of the target mRNA or inhibit its translation by pairing with the specific base of target mRNA and thus play a role in post-transcriptional regulation. Multiple miRNAs can jointly regulate the same target gene, and multiple target genes are capable of interacting with the same miRNA. By regulating the level of mRNA transcription, miRNA controls the amount of protein synthesis, thus regulating the occurrence and development of cardiovascular diseases. Once the specific miRNA is screened out, the target gene may be predicted. Furthermore, cardiomyocytes of miRNA inhibition and overexpression, following gene transfer using the miRNA mimic and miRNA inhibitors methods, may be cultured *in vitro* to explore the relationship between the miRNA and target genes in cardiomyocytes, which the authors believe should be studied further. The specific miRNAs found in the present study are the key to the further study of miRNA function.

## Figures and Tables

**Table I:** Purity of RNA in NC, AS and CS models.

Sample ID	OD_260/280_ (ratio)	OD_260/230_ (ratio)	Concentration, ng/μl	Volume, μl	Quantity, ng	QC result (pass/fail)
NC1	2.03	2.24	1,063.73	15	15,955.95	Pass
NC2	2.07	2.22	1,589.88	25	39,747.00	Pass
AS	2.06	2.22	1,533.42	50	76,671.00	Pass
CS	2.09	2.14	1,376.56	15	20,648.40	Pass

NC, normal control; AS, acute stress; CS, chronic stress; OD, optical density; QC, quality control.

**Table II:** Upregulated miRNA in the AS and CS models.

A, Upregulated miRNA (AS).		

Name	ID	Ratio scale slide 1 (AS/NC1)
rno-miR-21	5740	1.584
rno-miR-132	10937	4.004
rno-miR-141	10946	1.732
rno-miR-19a	10997	1.627
rno-miR-221	11022	1.600
rno-miR-223	11024	2.198
rno-miR-31	11052	1.504
rno-miR-32	11053	1.716
rno-miR-379	11093	1.669
rno-miR-129	11200	1.844
rno-miR-322	11225	1.524
rno-miR-336	11266	1.612
rno-miR-201	13176	1.679
rno-miR-376b-3p	14304	1.725
rno-miR-382	14307	2.007
rno-miR-147	17411	1.732
rno-miR-21	17896	1.697
rno-miR-139-5p	27542	1.618
rno-miR-34b	29153	1.693
rno-miR-674-3p	31053	1.503
rno-miR-34c	32772	1.882
rno-miR-219-5p	42509	1.701
rno-miR-291a-3p	42595	2.026
rno-miR-20b-5p	42640	1.730
rno-miR-296	42713	1.997
rno-miR-324-3p	42719	1.602
rno-miR-347	42763	1.907
rno-miR-451	42866	1.548
rno-miR-20a	42876	2.142
rno-miR-107	46629	1.566
rno-miR-20a	46793	1.639
rno-miR-375	46918	1.536

B, Upregulated miRNA (CS).		

Name	ID	Ratio scale slide 2 (CS/NC2)

rno-miR-141	10946	2.128
rno-miR-182	10975	1.528
rno-miR-194	10988	1.838
rno-miR-377	11091	1.579
rno-miR-448	11113	2.067
rno-miR-351	11235	1.636
rno-miR-344-3p	11268	2.615
rno-miR-381	14306	1.686
rno-miR-382	14307	1.676
rno-miR-200c	17427	1.726
rno-miR-877	30033	2.082
rno-miR-25	42481	2.027
rno-miR-219-5p	42509	1.519
rno-miR-671	42525	2.098
rno-miR-330	42606	2.212
rno-miR-615	42690	2.269
rno-miR-296	42713	2.231
rno-miR-218	42815	1.923
rno-miR-219-2-3p	42834	2.271
rno-miR-471	42916	2.519

NC, normal control; AS, acute stress; CS, chronic stress.

**Table III:** Downregulated miRNA AS and CS models.

Downregulated miRNA(AS)		

Name	ID	Ratio scale slide 1 (AS/NC1)
rno-miR-146a	10952	0.6450
rno-miR-184	10978	0.6700
rno-miR-300-3p	11221	0.6230
rno-miR-325-5p	11226	0.6000
rno-miR-329	11227	0.4570
rno-miR-341	11229	0.6137
rno-miR-297	11262	0.4580
rno-miR-344-3p	11268	0.6310
rno-miR-338	17825	0.5270
rno-miR-667	28944	0.6034
rno-miR-708	29190	0.2780
rno-miR-877	30033	0.5100
rno-miR-761	32608	0.3230
rno-miR-129	42467	0.5560
rno-miR-204	42502	0.5240
rno-miR-296	42528	0.6610
rno-miR-196a	42538	0.6040
rno-miR-342-5p	42576	0.4370
rno-miR-466c	42586	0.4200
rno-miR-330	42606	0.6210
rno-miR-30b-3p	42626	0.4800
rno-miR-878	42645	0.6120
rno-miR-490	42703	0.4800
rno-miR-325-3p	42706	0.4530
rno-miR-294	42707	0.6310
rno-miR-34c	42767	0.5980
rno-miR-665	42770	0.3530
rno-miR-150	42802	0.5930
rno-miR-300-5p	42826	0.5880
rno-miR-135a	42839	0.2680
rno-miR-125b	42845	0.6360
rno-miR-743b	42864	0.6190
rno-miR-330	42875	0.4010
rno-miR-551b	42917	0.4400
rno-miR-466b	42933	0.3010
rno-miR-742	42963	0.6440

Downregulated miRNA (CS)		

Name	ID	Ratio Scale Slide 2 (CS/NC2)

rno-miR-9	4040	0.5790
rno-miR-21	5740	0.5540
rno-miR-130a	10138	0.6440
rno-miR-146b	10306	0.6080
rno-miR-136	10943	0.3230
rno-miR-142-3p	10947	0.4850
rno-miR-146a	10952	0.6090
rno-miR-193	10986	0.5680
rno-miR-204	11005	0.4770
rno-miR-31	11052	0.3610
rno-miR-33	11062	0.5510
rno-miR-363	11077	0.4930
rno-miR-341	11229	0.6460
rno-miR-297	11262	0.5910
rno-miR-10a-5p	13485	0.4630
rno-miR-488	17316	0.5520
rno-miR-147	17411	0.5190
rno-miR-425	17608	0.6640
rno-miR-338	17825	0.6350
rno-miR-142-5p	19015	0.6220
rno-miR-106b	19582	0.6570
rno-miR-199a-5p	19590	0.5520
rno-miR-10a-3p	28019	0.5170
rno-miR-872	28250	0.6700
rno-miR-144	29802	0.6640
rno-miR-761	32608	0.5430
rno-miR-129	42467	0.6670
rno-miR-29b-1	42479	0.6100
rno-miR-136	42512	0.4920
rno-miR-338	42592	0.5560
rno-miR-325-3p	42706	0.6570
rno-miR-150	42802	0.5740
rno-miR-135a	42839	0.1210
rno-miR-330	42875	0.6280
rno-miR-466b	42933	0.3620

NC, normal control; AS, acute stress; CS, chronic stress.

**Table IV:** Upregulated miRNA in the AS and CS models.

Name	ID	Ratio scale slide 1(AS/NC1)	Ratio scale slide 1(CS/NC2)
rno-miR-141^a^	10946	1.732	2.128
rno-miR-382^a^	14307	2.007	1.676
rno-miR-219-5p^a^	42509	1.701	1.519
rno-miR-296^a^	42713	1.997	2.231

a significant upregulation in the AS and CS models.

AS, acute stress; CS, chronic stress; NC, normal control.

**Table V:** Downregulated miRNA in the AS and CS models.

Name	ID	Ratio scale slide 1 (AS/NC1)	Ratio scale slide 1 (CS/NC2)
rno-miR-135a^a^	42839	0.268	0.121
rno-miR-466b^a^	42933	0.301	0.362
rno-miR-146a	10952	0.645	0.609
rno-miR-341	11229	0.614	0.646
rno-miR-338	17825	0.527	0.635
rno-miR-761	32608	0.323	0.543
rno-miR-129	42467	0.556	0.667
rno-miR-325-3p	42706	0.453	0.657
rno-miR-150	42802	0.593	0.574
rno-miR-330	42875	0.401	0.628
rno-miR-297	11262	0.458	0.591

a significant downregulation in AS and CS.

AS, acute stress; CS, chronic stress; NC, normal control.
